# Ease pediatric emergency department crowding in Switzerland with high-quality telephone triage: a prospective multicenter study

**DOI:** 10.3389/fped.2025.1634841

**Published:** 2025-09-09

**Authors:** Krisztina Schmitz-Grosz, Carsten Sommer-Meyer, Stéphanie van der Lely, Siro Fritzmann, Georg Staubli, Eva Berger-Olah

**Affiliations:** ^1^ Medgate, Basel, Switzerland; ^2^ Pediatric Emergency Department and Children’s Research Centre, University Children’s Hospital Zurich, Zurich, Switzerland

**Keywords:** Kids Line, telemedicine, telephone triage, pediatric emergency department, quality, pediatric patients

## Abstract

**Introduction:**

This is the first study evaluating the picture of a pediatric telephone triage service's (PTTS) quality from the hospital, telemedical, and patient perspective, to provide a deeper understanding of its contribution to the relief of pediatric emergency burden.

**Methods:**

We conducted a prospective multicenter study from April 3 to May 15, 2023. All calls to the Medgate Kids Line of six hospitals providing pediatric emergency care in German-speaking Switzerland were included. Following telemedical counselling, patients were advised to visit a pediatric emergency department (PED) or a primary care provider (PCP) or were treated telemedically by the Kids Line team. Patients presenting to participating PEDs after calling were evaluated by a hospital triage specialist (HTS) to define telemedical triage's appropriateness [appropriate triage, undertriage (safety), overtriage (efficiency); hospital perspective]. Only PED presentations evaluated as undertriage or overtriage were peer-reviewed (telemedical perspective), while appropriate triages were adopted. Additionally, patients’ intention, adherence and satisfaction were assessed.

**Results:**

We included 4,061 calls. 24.9% cases were advised to go to a PED, 20.7% to a PCP, and 54.3% were allocated to telemedicine. HTSs evaluated 556 cases. The PTTS appropriately triaged 78.2% of cases according to the hospital perspective (undertriage: 8.1%; overtriage: 13.7%). After telemedical peer-review overall appropriateness was 91.7% (undertriage: 3.8%; overtriage: 4.5%). 606 patients provided feedback. Without PTTS, 76.9% would have consulted face-to-face medical care (PED: 60.6%). Adherence to triage recommendation was mostly high (PED: 84.1%; PCP: 23.3%; Telemedicine: 83.5%). Net promoter score was high (48.5).

**Conclusion:**

This PTTS (>100,000 calls/year) based on clinical expertise and guidelines is appropriate, safe, efficient, and patient-satisfactory and prevents a considerably high percentage of patients from visiting a PED. While patient adherence to triage recommendations “PED” and “Telemedicine” was high, lower adherence to PCP referrals might be explained by deviations in parents' perception of acuity, and/or limited PCP availability (at out-of-office hours). Triage appropriateness varied across perspectives. Incorporating such high-quality PTTSs into further regions of Switzerland may help alleviate the burden on the healthcare system.

## Introduction

The increasing burden on hospitals and emergency departments (EDs) due to overcrowding has become a critical global healthcare challenge (1), which has been linked to delayed care, diminished quality of care and increased costs (1–5). Unnecessary pediatric ED (PED) visits of parents with children having mild illness and minor injury significantly contribute to the above-mentioned problem (6–8). As previous studies showed, uncertainty and concerns regarding the severity of their child's condition, as well as factors like proximity to their residence and after-hours availability, frequently lead to low-acuity PED visits (6, 7, 9–11). Safe and efficient pediatric telephone triage services (PTTS) with or without telemedical treatment have been reported to be appropriate gatekeepers for healthcare resources (12). Globally, there are numerous PTTSs that aim to relieve ED burdens, however, the ranges for efficiency and safety vary highly (13–15) [e.g., with type of the triage/telemedical protocols (14, 16), applied evaluation approaches]. There are also concerns that the low barriers to access telemedicine may lead to overuse of healthcare services by patients who would not otherwise seek medical care (17). Consequently, it is essential to systematically analyse such services' quality from different perspectives. Such comprehensive evaluations of PTTSs are rather unexplored but may help to implement strategies to improve outcomes. Therefore, this study aimed to be the first to capture a comprehensive, multi-perspective picture of a PTTS's quality by considering a wide range of key aspects, thereby providing a deeper understanding of its contribution to the relief of PED crowding. These aspects included the appropriateness of triage dispositions (TDs) (safety/efficiency) from a hospital perspective (relying on general impressions from in-person visits), and from a peer-reviewed telemedical standpoint, as well as patient behaviour (e.g., primary intention and adherence to telemedical recommendations) and satisfaction.

## Materials and methods

We performed a prospective multicenter study from April 3 to May 15, 2023. The study was classified as a quality assurance project (Project-ID Req-2023-00282) by the local ethics committee (Ethikkommission Nordwest- und Zentralschweiz) and conducted according to the Declaration of Helsinki.

### Inclusion-/exclusion criteria

All eight Kids Line PEDs were considered to participate in the study, but two declined due to a lack of resources and organizational capacity. Consequently, we included all calls concerning medical advice for children aged from one day to 18 years to the Kids Line of the six participating hospitals (University Children's Hospital Zurich, University Children's Hospital Basel, Children's hospital of Aarau, Baden, Lucerne, and Winterthur) during the study period. We excluded calls with incomplete records [missing coded TD, International Classification of Primary Care (ICPC)-2 Code(s), age, or sex].

### The Kids Line

The Kids Line is a close cooperation between Medgate and several PEDs in German-speaking Switzerland. It provides telemedical advice to patients or their guardians 24/7 (language: German; >100,000 consultations in the first operation year). Consultations are conducted by agents [specifically pediatric nurses (all with many years of practical experience), pediatricians or non-pediatricians] trained and licensed in telemedicine, and include the application of internal pediatric guidelines based on national/international standards. Regular training sessions and quality controls are performed for all agent qualification groups. The primary Kids Line goal is to prevent parents and their children from unnecessary ED consultations and significantly relieve the children's hospitals' ED burdens through high-quality telephone triage and medical advice. Furthermore, the aim is to expand the Kids Line to additional Swiss regions, such as the French and Italian-speaking parts, however, key challenges are the variation in organizational structures across regions and cantons, and the coverage in multiple languages. See Supplementary Information 1 for information on Medgate and the Swiss healthcare system.

### Telemedical consultation: phase 1

**Figure 1:**
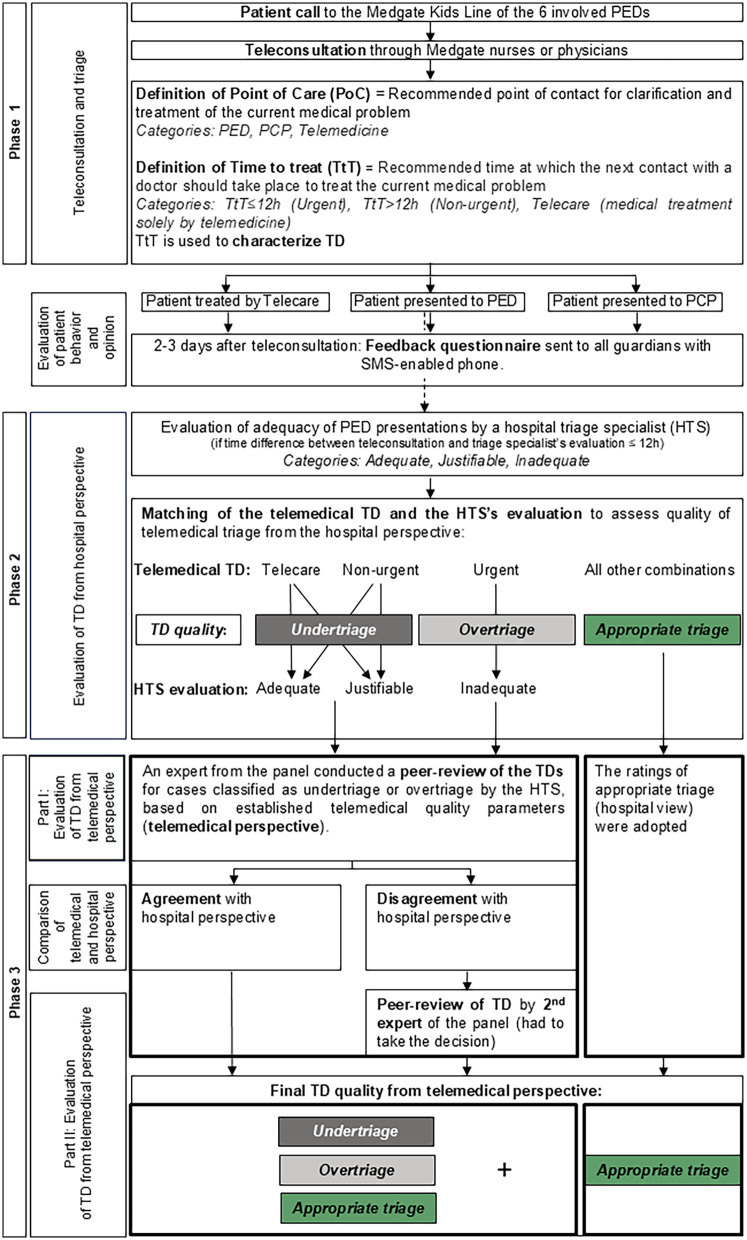
Study design and procedure flow chart. HTS, hospital triage specialist; PCP, primary care provider; PED, pediatric emergency department; PoC, point of care; TD, triage disposition; TtT, time to treat.

The telemedical consultations, were conducted as a first priority by pediatric nurses and pediatricians during day-time (07:00–22:59). If unavailable, non-pediatricians conducted the calls (second priority). Night shifts were covered by pediatricians and non-pediatricians. Advice for Point of Care (PoC) was either *PED, primary care provider (PCP) or Telemedicine* and for Time to Treat (TtT) *Urgent* (should be seen within 12 h by a physician)*, Non-urgent* (can be seen in >12 h by a physician) or *Telecare* (medical treatment solely by telemedicine, Figure 1). TtT was used to define TD.

### Evaluation of TD from hospital perspective: phase 2

For patients presenting to a participating PED after calling the Kids Line (independent of TD), a hospital triage specialist [HTS; educated in Australasian Triage assessment (18–20)] evaluated the adequacy of PED presentation at first contact as adequate (correct TtT and PoC), justifiable (correct TtT but PED triage not needed, PCP triage would be more appropriate; possible reasons: fear/concerns of parents or external circumstances such as non-reachable PCP in the medically required time frame), or inadequate (wrong/suboptimal TtT). HTSs were trained in this categorization. To identify study patients at the PED, Kids Line patients were instructed to inform hospital staff about their use of the service upon visiting, and HTSs also inquired about any prior Kids Line consultation.

The telemedical TD and the HTS's evaluation of PED presentation adequacy was matched to define appropriateness of TD, safety/undertriage and efficiency/overtriage from the hospital perspective (Figure 1). Cases in which the time interval between teleconsultation and the HTS's evaluation exceeded 12 h were excluded due to possible and expected changes in patients' health condition.

### Evaluation of TD from telemedical perspective: phase 3

Cases deemed as undertriage or overtriage by the HTS underwent peer-review by an expert panel (telemedical specialists, pediatricians with telemedical expertise; each with 5–15 years of telemedical experience). The panel evaluated these cases' TD from the telemedical perspective by listening to the recorded teleconsultation. The TD was rated as appropriate, undertriage or overtriage using a standardized, company-intern evaluation based on the Assessment of Quality in Telephone Triage (21). See Figure 1 for information on the peer-review process. A subgroup analysis was conducted focusing on the safety of patients classified as undertriage (Supplementary Information 2).

### Patient feedback

2–3 days after teleconsultation, all guardians (or the patients) received a feedback questionnaire on their mobile (Supplementary Information 3). The percentage of patients who intended to stay at home if the Kids Line did not exist was used to estimate potential increases in healthcare usage. Furthermore, patient's behavior following teleconsultation was aligned with the recommended PoC to calculate patient adherence. The likelihood to recommend the Kids Line service to others (0 not at all–10 very likely) was used to calculate the net promoter score (NPS), a widely used score to measure patient satisfaction. Survey respondents were categorized into “promoters” [likelihood to recommend (LTR): 9–10], “passives” (LTR: 7–8) or “detractors” (LTR: 0–6) and NPS was calculated by subtracting the percentage of detractors from the percentage of promoters (range −100 to 100). Patients without SMS-enabled phone were excluded from this analysis.

### Data processing

To categorize reason for encounter (RFE), the combination of ICPC-2 Codes allocated to each case were assigned to one of eight RFE-groups (defined considering this study population's most prevalent symptoms).

The time-point of the calls was categorized (Working day: Monday–Friday, Non-working day: Saturday/Sunday/three public holidays; day-shift: 07:00–22:59, night-shift: 23:00–06:59).

### Statistical analysis

Data processing and statistical analyses were performed using Python (Version 3.10.9) and RStudio (Version 1.1.456, U.S.A.). Categorical data are described as counts and percentages. Continuous variables are presented with median and interquartile range (IQR). Normal distribution was tested using Shapiro–Wilk test and by visual inspection of QQ-plots.

To identify explanatory variables of TD, TD quality (appropriate triage/undertriage/overtriage) or agreement/disagreement between raters, we used Kruskal Wallis rank sum tests, Pearson Chi-squared tests, or Fisher's Exact tests [for count data with simulated *p*-value (based on 2,000 replicates)]. Mosaic plots and independence tests using Pearson residuals were used to investigate relative frequencies and associations between categorical variables (R packages vcd, gtsummary). To investigate this PTTS's potential in relieving PEDs, flow from patient intention to PoC (PED/non-PED) and adherence was analyzed using a Sankey Flow diagram (patients with intention “I do not know” were excluded).

## Results

### Patient characteristics

**Figure 2:**
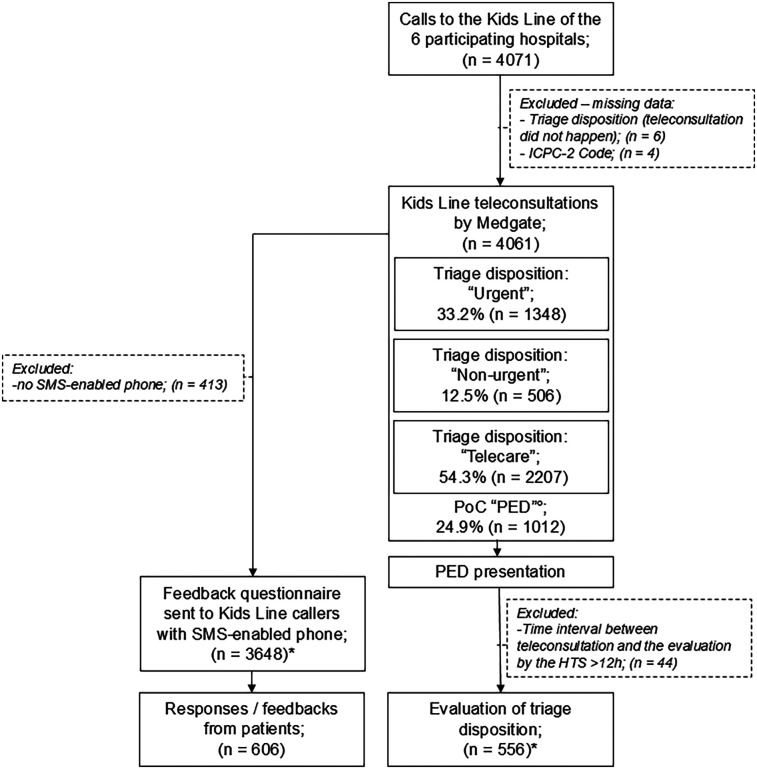
Flow diagram of patient cohort. HTS, hospital triage specialist; ICPC-2 Code, international classification of primary care-2 Code; PED, pediatric emergency department; PoC, point of Care.°The 1,012 patients with PoC “PED” are also included in the above-listed triage disposition groups. *A patient can be listed in both groups.

4,061 Kids Line calls were included (Figure 2, baseline/call characteristics: Table 1). Calls concerning infants and toddlers made up the largest proportion (79% below 6 years). Infections (e.g., gastrointestinal complaints/fever) were the most frequent reason for consultation, regardless of age category, with the exception of trauma, which was the predominant cause in children >6 years.

**Table 1:** (a) Baseline characteristics of patients who consulted the Kids Line of the six participating PEDs (*n* = 4,061) during the study period. (b) Characteristics of the corresponding calls. Data are represented as count and percentage or median (interquartile range), where appropriate. Percentages in parentheses indicate the proportion of each subgroup within the overall cohort (column percentages).

(a) Baseline characteristics	Total (*n* = 4,061)
Age, years; median (IQR)	2.0 (0.0–5.0)
Age categories	
[0–4 months]; *n* (%)	354 (8.7%)
[5 months–1 year); *n* (%)	784 (19.3%)
[1 year–6 years); *n* (%)	2,089 (51.4%)
[6 years–12 years); *n* (%)	653 (16.1%)
[12 years–18 years); *n* (%)	181 (4.5%)
Sex	
Female; *n* (%)	1,892 (46.6%)
Male; *n* (%)	2,169 (53.4%)
Reason for encounter group	
Gastrointestinal complaints; *n* (%)	868 (21.4%)
Fever; *n* (%)	728 (17.9%)
Trauma; *n* (%)	696 (17.1%)
Respiratory complaints; *n* (%)	667 (16.4%)
Worries; *n* (%)	189 (4.7%)
Ear pain; *n* (%)	165 (4.1%)
Dermatological complaints; *n* (%)	239 (5.9%)
Other^a^; *n* (%)	509 (12.5%)
(b) Call characteristics	Total (*n* = 4,061)
Day of call	
Working day; *n* (%)	2,062 (50.8%)
Non-working day; *n* (%)	1,999 (49.2%)
Time of call	
Day shift: 07:00–22:59; *n* (%)	3,358 (82.7%)
Night shift: 23:00–06:59; *n* (%)	703 (17.3%)
Qualification of agent	
Pediatric nurses; *n* (%)	2,218 (54.6%)
Pediatricians; *n* (%)	323 (8.0%)
Non-pediatricians; *n* (%)	1,520 (37.4%)

^a^ The reason for encounter group “Other” includes patients with allergies, chest pain, intoxication, neurological problems, urinary tract infection, restlessness, conjunctivitis, or general health issues.

### Triage disposition

Recommended PoC was “PED” in 24.9%, “PCP” in 20.7% and “Telemedicine” in 54.3%. Considering TtT, 33.2% of the patients were categorized as TD “urgent”, 12.5% as “non-urgent”, and most cases (54.3%) could be allocated to “telecare”. Patient age, RFE-group, day/time of call, and agent qualification was significantly associated with TD distribution (Supplementary Table 1).

**Figure 3:**
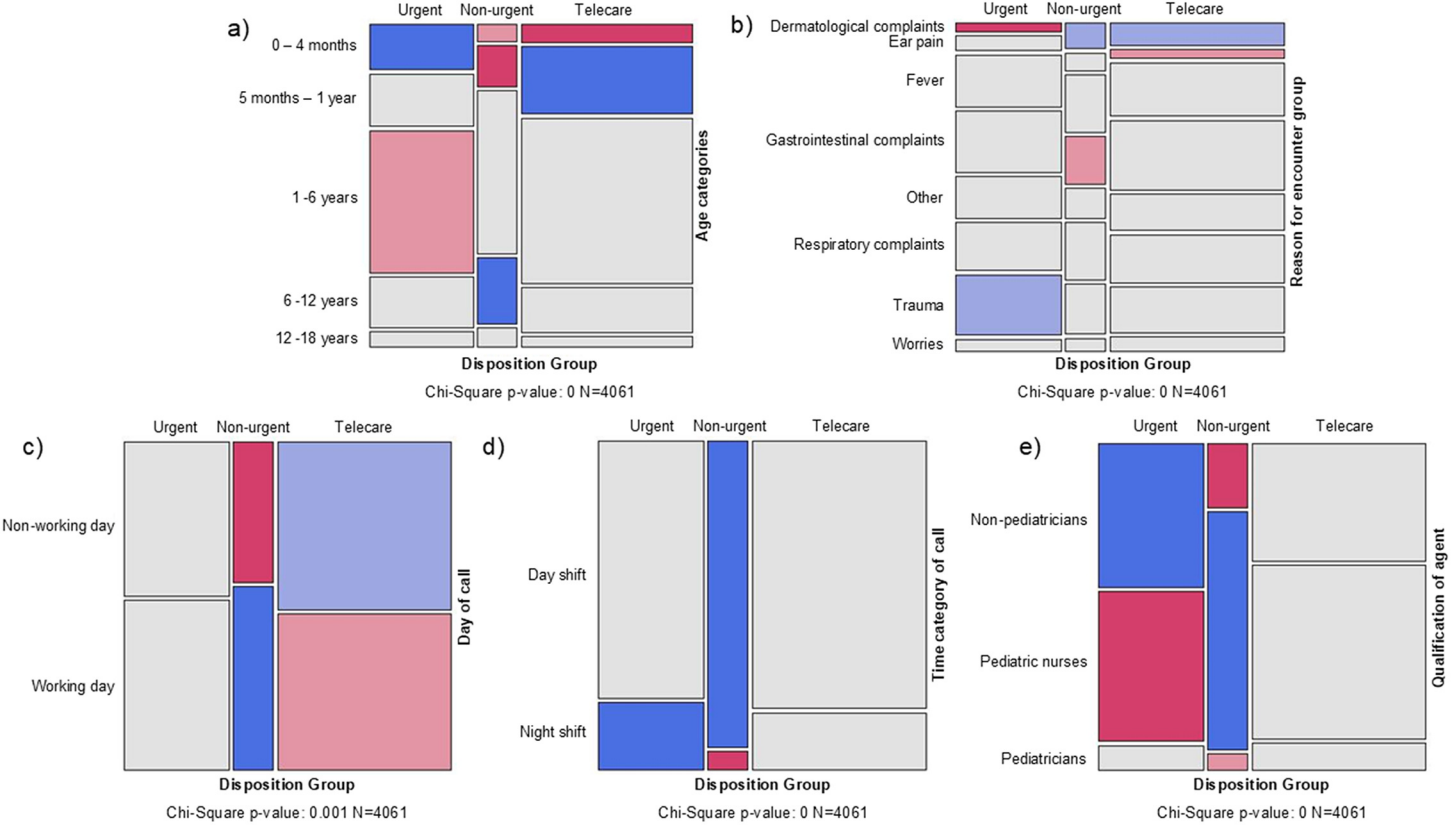
Mosaic plots, indicating positive and negative associations between each triage disposition and patient characteristics or call characteristics (based on a Pearson chi-squared test of independence, *p*-value reported in plot, *n* = 4,061). The area of cells is proportional to the frequency of elements in a contingency table. The color of the cells refers to the sign and magnitude of the respective Pearson residuals (blue indicates a significantly higher value than expected if the data were random and red indicates a significantly lower value than expected [dark blue/dark red: significant residual at 99% significance level, light blue/light red: 90% significance level)]. **(a)** Triage dispositions by age categories of the patients, **(b)** Triage disposition by reason for encounter (RFE) groups. RFE-group—“Other” includes patients with allergies, chest pain, intoxication, neurological problems, urinary tract infection, restlessness, conjunctivitis, or general health issues; **(c)** Triage disposition by day of call (non-working and working day); **(d)** Triage disposition by time category of call (day and night shift); **(e)** Triage disposition by qualification of agent (pediatric nurses/pediatricians/non-pediatricians).

In patients aged under 4 months, TD “urgent” was more frequent, while TDs “non-urgent” and “telecare” were less likely. TD “non-urgent” was also less prevalent in patients aged between 5 months and 1 year, however, telemedical treatment was more often observed in this group. For those aged between 1 and 6 years, TD “urgent” was less common, while TD “non-urgent” was more prevalent in patients aged between 6 and 12 years (Figure 3a). Our results also indicated that patients with dermatological complaints were less likely to receive TD “urgent” but more likely got TDs “non-urgent” or “telecare”, respectively. In trauma patients, TD “urgent” was more likely, while TD “non-urgent” was less likely in patients with gastrointestinal complaints. Telecare was not commonly recommended in pediatric patients with ear pain (Figure 3b). Calls during non-working days more likely resulted in TD “telecare”, whereas the opposite was observed for working days (Figure 3c). During the night TD “urgent” was more likely and TD “non-urgent” was less common, while TD “non-urgent” was more common during the day (Figure 3d). Pediatric nurses were more prone to classify cases as “non-urgent”, while TD “urgent” was less often. On the other hand, non-pediatricians were more prone to classify cases as “urgent”, while TD “non-urgent” was less often (Figure 3e).

### TD quality—hospital perspective

**Figure 4:**
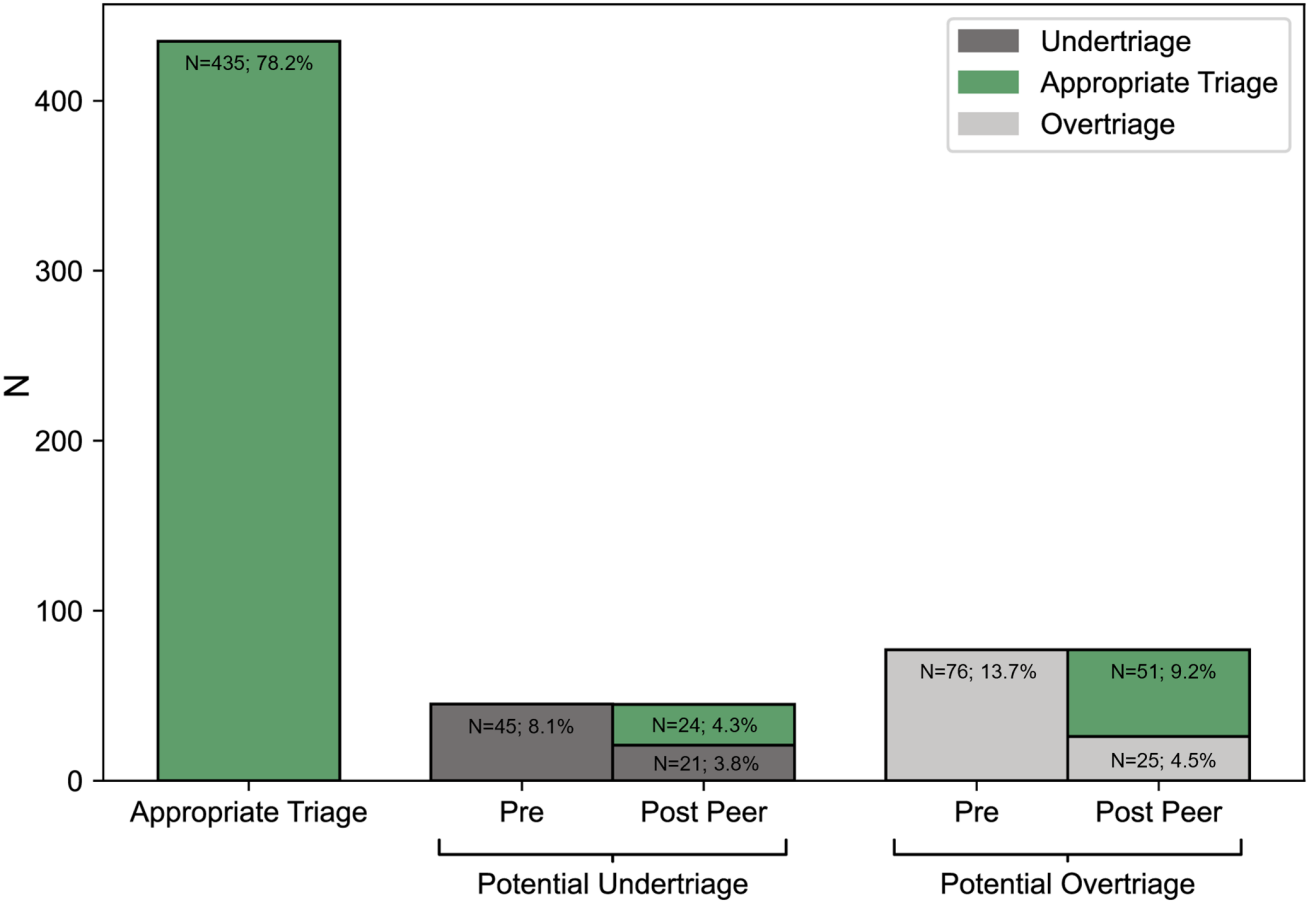
Number of cases that were evaluated as appropriate triage (*n* = 435, 78.2%), potential undertriage (*n* = 45, 8.1%) and potential overtriage (*n* = 76, 13.7%) by the hospital triage specialist (Hospital perspective; Pre Peer). The peer-review by the expert panel of the cases deemed as undertriage and overtriage revealed 75 cases of appropriate triage (13.5%, 75 out of 556 patients), 21 cases of potential undertriage (3.8%) and 25 cases of overtriage (4.5%; Telemedical perspective, Post Peer). Evaluations where the hospital triage specialist supported the triage disposition with the rating “appropriate” were adopted for the telemedical perspective (total rate of appropriate triages: 13.5% + 78.2%=91.7%).

HTSs evaluated 556 PED presentations (Figure 2, Supplementary Table 2 for the baseline/call characteristics). The telemedical TD of these patients was “urgent” in 87.6%, “non-urgent” in 2.9%, and 9.5% were previously advised to be treated by telecare. Time interval between teleconsultation and HTS evaluation was 1.5 h (IQR: 1.0–2.1 h). The HTS' rating revealed appropriate telephone triage in 78.2% (undertriage: 8.1%—no hospitalisation, overtriage: 13.7%; Figure 4; Pre-Peer). Patient age, sex, RFE-group, time category of the call and day of the call had no significant influence on TD quality (appropriate triage/undertriage/overtriage), while agent qualification (pediatric nurses, pediatricians, non-pediatricians) did have a significant influence (Table 2). All groups were rated as having a high percentage of appropriate triages. Although the overtriage rates of pediatric nurses (8.3%) but also pediatricians (13.7%) were lower compared to non-pediatricians (18.6%), their rates of undertriage were higher.

**Table 2:** Patient and call characteristics of the cases evaluated as appropriate triage, undertriage or overtriage and results of the statistical tests showing the association of the respective variables with quality of triage disposition, when evaluated from the hospital perspective by the hospital triage specialist (*n* = 556). Data are represented as count and percentage or median (interquartile range), where appropriate. Percentages in parentheses indicate the proportion of each outcome within the respective subgroup (row percentages).

	Appropriate triage (*n* = 435)	Undertriage (*n* = 45)	Overtriage (*n* = 76)	*p*-value
Age, years; median (IQR)	2.0 (0.0–5.0)	2.0 (1.0–5.0)	2.0 (1.0–5.0)	0.59
Age categories				0.23
[0–4 months]; *n* (%)	58 (89.2%)	3 (4.6%)	4 (6.2%)	
[5 months–1 year); *n* (%)	77 (80.2%)	8 (8.3%)	11 (11.5%)	
[1 year–6 years); *n* (%)	199 (73.7%)	28 (10.4%)	43 (15.9%)	
[6 years–12 years); *n* (%)	77 (81.9%)	4 (4.3%)	13 (13.8%)	
[12 years–18 years); *n* (%)	24 (77.4%)	2 (6.5%)	5 (16.1%)	
Sex				0.51
Female	212 (80.0%)	18 (6.8%)	35 (13.2%)	
Male	223 (76.6%)	27 (9.3%)	41 (14.1%)	
Reason for encounter group				0.22
Gastrointestinal complaints; *n* (%)	89 (73.0%)	10 (8.2%)	23 (18.9%)	
Fever; *n* (%)	73 (82.0%)	3 (3.4%)	13 (14.6%)	
Trauma; *n* (%)	97 (78.2%)	10 (8.1%)	17 (13.7%)	
Respiratory complaints; *n* (%)	72 (80.0%)	9 (10.0%)	9 (10.0%)	
Worries; *n* (%)	14 (77.8%)	3 (16.7%)	1 (5.6%)	
Ear pain; *n* (%)	23 (88.5%)	1 (3.8%)	2 (7.7%)	
Dermatological complaints; *n* (%)	9 (56.3%)	4 (25.0%)	3 (18.8%)	
Other^a^; *n* (%)	58 (81.7%)	5 (7.0%)	8 (11.3%)	
Day of call				0.08
Working day; *n* (%)	208 (76.2%)	19 (7.0%)	46 (16.8%)	
Non-working day; *n* (%)	227 (80.2%)	26 (9.2%)	30 (10.6%)	
Time category of call				0.16
Day shift: 07:00–22:59; *n* (%)	355 (78.5%)	40 (8.8%)	57 (12.6%)	
Night shift: 23:00–06:59; *n* (%)	80 (76.9%)	5 (4.8%)	19 (18.3%)	
Qualification of agent				<0.001*
Pediatric nurses; *n* (%)	191 (79.3%)	30 (12.4%)	20 (8.3%)	
Pediatricians; *n* (%)	38 (74.5%)	6 (11.8%)	7 (13.7%)	
Non-pediatricians; *n* (%)	206 (78.0%)	9 (3.4%)	49 (18.6%)	

^a^ The Reason for encounter group “Other” includes patients with allergies, chest pain, intoxication, neurological problems, urinary tract infection, restlessness, conjunctivitis, or general health issues.

Significant differences are marked: *p < 0.05.

### TD quality—telemedical perspective

Peer-review comprised 45 cases of undertriage and 76 cases of overtriage. Overall, the rating from the telemedical perspective indicated appropriate triage in 91.7% (undertriage: 3.8%, overtriage: 4.5%) (Figure 4; Post-Peer). Of all the variables, only agent qualification (pediatric nurses, pediatricians, non-pediatricians) was significantly associated with TD quality (Table 3). According to the telemedical perspective, all groups appropriately triaged a high percentage of patients (>90%). While the overtriage rate of pediatric nurses (2.5%), as well as that of pediatricians (2.0%), was lower compared to non-pediatricians (6.8%), their undertriage rates were higher. The level of agreement/disagreement between the two perspectives significantly depended on RFE-group (Supplementary Table 3).

**Table 3:** Patient and call characteristics of the cases evaluated as appropriate triage, undertriage or overtriage and results of the statistical tests showing the association of the respective variables with quality of triage disposition, when evaluated from the telemedical perspective (*n* = 556). Data are represented as count and percentage or median (interquartile range), where appropriate. Percentages in parentheses indicate the proportion of each outcome within the respective subgroup (row percentages).

	Appropriate triage (*n* = 510)	Undertriage (*n* = 21)	Overtriage (*n* = 25)	*p*-value
Age, years; median (IQR)	2.0 (0.0–5.0)	1.0 (1.0–5.0)	2.0 (1.0- 8.0)	0.29
Age categories				0.18
[0–4 months]; *n* (%)	62 (95.4%)	1 (1.5%)	2 (3.1%)	
[5 months–1 year); *n* (%)	92 (95.8%)	3 (3.1%)	1 (1.0%)	
[1 year–6 years); *n* (%)	241 (89.3%)	14 (5.2%)	15 (5.6%)	
[6 years–12 years); *n* (%)	89 (94.7%)	2 (2.1%)	3 (3.2%)	
[12 years–18 years); *n* (%)	26 (83.9%)	1 (3.2%)	4 (12.9%)	
Sex				0.65
Female	246 (92.8%)	9 (3.4%)	10 (3.8%)	
Male	264 (90.7%)	12 (4.1%)	15 (5.2%)	
Reason for encounter group				0.15
Gastrointestinal complaints; *n* (%)	115 (94.3%)	2 (1.6%)	5 (4.1%)	
Fever; *n* (%)	79 (88.8%)	3 (3.4%)	7 (7.9%)	
Trauma; *n* (%)	113 (91.1%)	4 (3.2%)	7 (5.6%)	
Respiratory complaints; *n* (%)	84 (93.3%)	4 (4.4%)	2 (2.2%)	
Worries; *n* (%)	18 (100.0%)	0 (0.0%)	0 (0.0%)	
Ear pain; *n* (%)	23 (88.5%)	1 (3.8%)	2 (7.7%)	
Dermatological complaints; *n* (%)	12 (75.0%)	4 (25.0%)	0 (0.0%)	
Other^a^; *n* (%)	66 (93.0%)	3 (4.2%)	2 (2.8%)	
Day of call				0.91
Working day; *n* (%)	249 (91.2%)	11 (4.0%)	13 (4.8%)	
Non-working day; *n* (%)	261 (92.2%)	10 (3.5%)	12 (4.2%)	
Time category of call				0.47
Day shift: 07:00–22:59; *n* (%)	411 (90.9%)	19 (4.2%)	22 (4.9%)	
Night shift: 23:00–06:59; *n* (%)	99 (95.2%)	2 (1.9%)	3 (2.9%)	
Qualification of agent				0.01*
Pediatric nurses; *n* (%)	222 (92.1%)	13 (5.4%)	6 (2.5%)	
Pediatricians; *n* (%)	46 (90.2%)	4 (7.8%)	1 (2.0%)	
Non-pediatricians; *n* (%)	242 (91.7%)	4 (1.5%)	18 (6.8%)	

^a^ The Reason for encounter group “Other” includes patients with allergies, chest pain, intoxication, neurological problems, urinary tract infection, restlessness, conjunctivitis or general health issues.

Significant differences are marked: *p < 0.05.

### Patient feedback

606 patients provided feedback (Figure 2, Supplementary Table 4). Without PTTS, 76.9% would have consulted face-to-face medical care (PED: 60.6%, PCP: 16.3%), 10.0% would have stayed at home, and 13.1% answered “I do not know”. Adherence to triage recommendation was high for patients with PoC “PED” (84.1%) and “Telemedicine” (83.5%), while patients with PoC “PCP” were the least likely to comply with the recommendation [23.3%; non-adhering patients opted for telemedicine (62.0%) or went to the PED (38.0%)].

**Figure 5:**
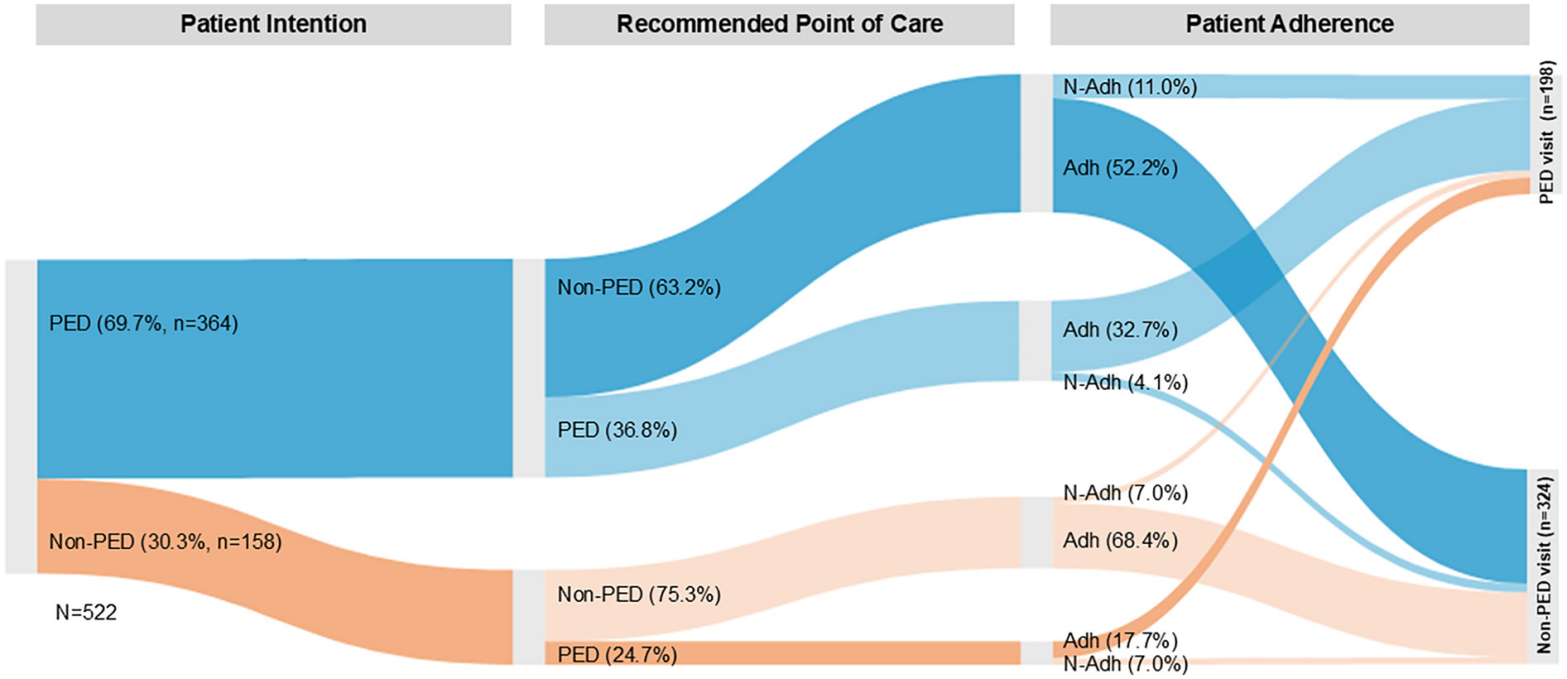
Sankey flow diagram of patient flow (*n* = 522) from patient intention to recommended point of care (categorized as PED/non-PED [PCP, telemedicine]) and adherence. Patients with intention “I do not know” were not included in this flow diagram (*n* = 79). The percentages shown in the “Point of Care” and “Adherence” sections indicate what proportion of patients in each intention group were referred to the various Points of Care and how they finally behaved. PED intention: Patient intended to visit a PED (blue streams), Non-PED intention: Patient did not intend to visit a PED (orange streams). Adherence (Adh): Patient adhered to the recommendation of visiting or not visiting a PED. Non-Adherence (N-Adh): Patient did not adhere to the recommendation of visiting or not visiting a PED. Values represent the percentage of patients at each stage of the process. The size (width) of each line presented in the diagram is proportional to the quantity of patients per group.

When not considering patients with intention “I don’t know”, the Sankey flow (Figure 5) indicated that 52.2% of the patients who initially intended a PED visit could be prevented from doing so, as they followed the advice of not visiting a PED. On the other hand, 17.7% of the patients with intention “non-PED” adhered to the recommendation of visiting a PED. An improvement in health condition was reported by 86.7% of the patients (10.8%: no change; 2.5%: deterioration). Among the patient group that adhered to PoC “Telemedicine”, 87.4% indicated an improved health condition. NPS was 48.5.

## Discussion

Despite the widespread use of PTTSs and their increasing popularity, comprehensive descriptions of the quality of such services remain scarce. The present findings provide valuable insights into the quality of a high-volume telemedicine center regarding critical aspects such as the appropriateness of the triaging of pediatric patients.

When TD of Kids Line patients that visited a PED was evaluated by experts listening to the teleconsultation (telemedical perspective), 91.7% of patients were appropriately prioritized based on clinical urgency. The low rates of undertriage (3.8%) and overtriage (4.5%) revealed high patient safety and efficiency, which represent crucial quality factors. Our results from the telemedical perspective align with a study from Israel, where physicians rated pediatric TDs of urgent nature based on recorded teleconsultations (appropriate triage: 92.0%, undertriage: 5.3%, overtriage: 2.7%) (14). Furthermore, Graversen et al. (2023) revealed undertriage in 3.7% and overtriage in 7.5%, when physicians listened to audio-recordings of randomly selected pediatric patient calls (22). Consequently, lower undertriage rates seem to be accompanied by higher overtriage rates. While our undertriage rate was low and comparable to other studies, none of our undertriaged cases required hospitalisation. Also the potential delays in PED presentation due to TD “Telecare” did not bear critical risks (no hospitalisations).

However, when triage was evaluated by HTSs, our results revealed appropriate triage in 78.2%, undertriage in 8.1%, and overtriage in 13.7%. The differences between the two perspectives might be partly attributed to cases that involved highly concerned guardians or RFEs such as gastrointestinal complaints, as these were often evaluated as more urgent by the telemedical perspective. Furthermore, changes in patients' health status could have affected the comparability (telemedical perspective: evaluation at time of teleconsultation; hospital perspective: at arrival at PED) despite the 12-h time limit between the ratings. Other studies evaluating TDs in hospitals revealed overtriage rates between 9.3% and 54% (15, 23, 24). Two studies used broader evaluation scopes, where patient history, findings from physical examinations, urgent needs for tests/therapy (23), or information from the ED course (24), were included. Our study demonstrated an overtriage rate in the lower range, making it a more effective service than most others. However, differences between studies should be interpreted with caution as the reported rates can vary due to different factors (e.g., with the methods used to assess triage quality). Undertriage rates were not included in the design of other studies investigating the hospital perspective in broad pediatric populations, as most studies exclusively evaluated PTTS' PED referrals.

A thorough evaluation of TD quality requires close attention to both the disposition itself and the factors that influence it. In this study we observed several significant influences on TD. Besides external factors (e.g., PCP unavailability), also age and RFE-group played a role in decision-making. Younger patients (<4 months) or those with trauma, more often required immediate PED referral, while in some older age categories or cases involving dermatological complaints TD “non-urgent” or “telecare” was more common. The finding that pediatric nurses were more prone to classify cases as “non-urgent”, while TD “urgent” was less often, could be attributed to the pediatric nurses' substantial practical experience, specialized training in pediatric emergency triage, in-depth knowledge of the involved hospitals, and differing educational backgrounds from those of physicians. All these factors may have contributed to the observed differences in TD. On the other hand, the finding that TD “urgent” was more prevalent in non-pediatricians reflected this group's tendency to be more cautious when triaging, perceiving cases as more urgent. This result might be explained by the fact that non-pediatricians had no specialization in pediatrics, in contrast to the other two groups. Similar to the night-shift, fewer TDs “non-urgent” were observed on non-working days. Overall (during day and night), >50% of the patients received TD “telecare”, which is comparably high compared to other PTTSs (25–28).

Previous studies also described factors associated with an unsafe telephone triage [i.e., infants (25), calls during nighttime (25), abdominal pain (29–32)]. In this study, we did not find a significant association between patient characteristics and under-/overtriage rates (Tables 2, 3). Nevertheless, as certain RFEs (e.g., respiratory complaints) showed a rather high rate of undertriage, triage professionals should pay extra attention when certain medical complaints are mentioned. Regarding call characteristics, however, agent qualification was found to be significantly associated with under-/overtriage rates, which was most likely a result of the different distribution of TDs. A tendency towards a more cautious and safe triaging approach, as observed in non-pediatricians, and the lack of a specialization in pediatrics seemed to lead to a higher percentage of overtriage (and less undertriage). The lower rate of overtriage observed among pediatricians and pediatric nurses indicated, however, high efficiency, and suggested a potential role in reducing PED overcrowding by ensuring more appropriate use of emergency resources. Although it was accompanied by a higher rate of undertriage, none of the undertriaged cases required hospitalization and no fatal outcomes occurred. Undertriage and overtriage are opposing aspects in the effort to achieve the most accurate triage possible.

The finding that the differences between agent qualification groups were relatively small and that only agent qualification was significantly associated with TD quality (no associations with patient characteristics or other call characteristics) may reflect a strength of Kids Line's triage protocols. The combination of clinical expertise, regular quality management, high-quality telemedical training provided to the medical professionals, and the application of internal guidelines tailored to pediatric care, likely represented essential elements that minimized the impact of factors typically associated with a higher risk of triage errors. This level of standardization appears to support consistent and reliable clinical decision-making. Previous studies also underscored the role of training in enhancing triaging (16) and highlighted that a standardized approach and targeted questioning could positively influence decision-making (33). However, although no significant effects were observed for the majority of the examined factors on TD quality, it cannot be ruled out that the sample size, particularly the relatively small counts for some combinations, was insufficient to detect such effects. This should be considered when interpreting our findings, and future research with larger samples is warranted to validate these results.

Regarding patient behaviour, this study found that 76.9% of the patients would have otherwise claimed medical consultation, predominantly in PED settings (60.6%). The fact that only 10% intended to stay at home without the Kids Line can alleviate concerns about healthcare over-usage. Similarly to other pediatric studies, patients with PoC “PED” or “Telemedicine” exhibited higher rates of adherence to teleconsultation recommendations compared to those with PoC “PCP” (25, 28, 34). Based on other studies, the reasons why patients were non-adherent could include changes in the child's condition, deviations in parents' perception of acuity, or misunderstandings (28, 35). Furthermore, limited PCP availability outside of regular office hours, which is a general issue affecting healthcare systems worldwide and is also significant in Switzerland, could explain this finding. To improve adherence to PCP recommendations, direct booking of PCP appointments during teleconsultations should be implemented to facilitate scheduling and enable adjustments to recommendations when PCPs are unavailable. In addition, these findings highlight the importance of enhancing patient education regarding the PCP visit. It is essential to clearly communicate the reasons for the recommended time to treat, advise patients on what signs to monitor before their appointment, and reassure them that they can contact the telemedical provider at any time if they have concerns about their health prior to the PCP visit. Alternatively, a timely telemedical follow-up could be arranged if the PCP is only available at a later timepoint. Adherence to the telemedical recommendations “PED” or “Telemedicine” was higher or comparable to other studies (14, 25, 28, 36, 37).

The fact that a significant percentage (52.2%, Figure 5) of the patients that initially planned a PED visit could be rerouted showed that telemedical guidance could mitigate non-urgent health concerns from escalating into PED visits. Our findings supported another study highlighting low-acuity PED use as a significant issue in Switzerland (7).

On the other hand, the finding that 17.7% of patients who initially did not intend to go to a PED but after triage according to medical guidelines adhered to PoC “PED”, demonstrated the ability in detecting unrecognized potential serious medical conditions. In general, our results indicated that patient flow could be well controlled with the Kids Line with a positive impact on patients' health (improvements in >85%). This large number and the high rate of appropriate triages confirmed that our service effectively and safely managed pediatric medical problems. Our service is a valuable gatekeeper with the potential to reduce healthcare costs, and burdens on patients and parents (12, 13). Our NPS of 48.5 represents a high level of patient satisfaction in the healthcare industry and is comparable to another telehealth study (38). Such a high NPS value reflects the quality of care provided and impacts reputation.

However, the study had several limitations. The primary limitation is that triage quality was evaluated only among patients who attended a participating PED, and that feedback could be obtained from only a subset of patients or caregivers. Focusing solely on the service's triage appropriateness for this patient subset may have introduced selection bias that limits the results' generalizability. Nonetheless, this approach provided a valuable, multi-faceted assessment of the PTTS's safety and efficiency (even if only for this specific patient subset), made possible by a unique collaboration between a high-volume telemedicine provider and several PEDs in German-speaking Switzerland. However, it is important that future studies enhance our understanding of triage quality of other patient/TD groups. Furthermore, the number of HTS evaluations indicated that TD quality was not assessed of all patients who have finally visited a PED. One possible reason could be that certain patients visited a non-participating PED in their area. Furthermore, since patient feedback was only available from a subset of patients, likely due to the unfortunate current trend of decreasing willingness to complete voluntary surveys, there may be a response bias that limits the transferability of the findings to a larger population. Although the collected insights played a key role in understanding the service's influence on patient behavior and satisfaction, it is important to note that previous studies have shown that patients may hesitate to disclose sensitive information—such as not following medical advice—especially when surveys are conducted by the telemedical center involved in their care (37). Therefore, involving independent centers may be reasonable. Furthermore, the one-time patient feedback impeded to assess long-term outcomes and potential further treatment locations. Another limitation of the study was that this multicenter study was conducted only in German-speaking Switzerland which might limit the findings' application to other regions/systems. Additionally, the peer-review panel included Medgate employees, which might have introduced bias. However, this was accepted as they possessed longstanding experience in telemedicine, which is hardly comparable in Switzerland.

## Conclusion

Our findings suggested that this large-volume PTTS (>100,000 calls/year) based on the combination of high-quality telemedical and clinical expertise offers an appropriate, safe, and efficient way of adequately allocating valuable healthcare resources, and thereby helps to alleviate PED crowding. Overall, the quality of TD was high, however, discrepancies were revealed when comparing evaluations from different perspectives (listening to recorded teleconsultations vs. including patient impression and appearance). While patient intention indicated minimal potential for healthcare over-usage, the high adherence to PED referrals and Telemedicine, as well as the high patient satisfaction further underscored this PTTS's value in meeting patient needs. The lower adherence to PCP referrals might be explained by deviations in parents' perception of acuity, and/or limited PCP availability (at out-of-office hours). Incorporating such high-quality PTTSs into further regions of Switzerland may help optimize pediatric patient flow and potentially reduce the burden on the healthcare system, patients, and their parents. However, further studies are needed to confirm these effects.

## Data Availability

The data supporting the findings of this study are available from the corresponding author, upon reasonable request and in accordance with the data security regulations of the company's regulatory compliance department. Requests to access the datasets should be directed to Krisztina Schmitz-Grosz, krisztina.schmitz-grosz@medgate.ch.
