# Supplementary material for: Ease pediatric emergency department crowding in Switzerland with high-quality telephone triage: a prospective multicenter study
Source: Front Pediatr. 2025 Sep 9;13:1634841. doi: 10.3389/fped.2025.1634841 (PMC12455728; doi:10.3389/fped.2025.1634841)
Supplement: Supplementary file 1 [file Supplementaryfile1.docx]

Supplementary Material

# Supplementary Information

**Supplementary Information 1**

**Information on the telemedical center Medgate**

Medgate is a private company that has been providing telemedicine services 24 hours a day, 365 days a year since 2000. Since then, more than 13 million teleconsultations have been conducted. The company operates internationally (Switzerland, Germany, and the Philippines), offering teleconsultations in German, French, Italian (Swiss national languages) or English. The main medical specialities include pediatrics and adult medicine. Since October 1, 2021, Medgate has been operating the Kids Line. A Kids Line telemedical consultation includes telemedical examinations such as telestatus or basic vital signs assessment (no video).

**Information on the Swiss healthcare system**

The Swiss healthcare system is based on a mandatory basic insurance, which is compulsory for all residents and is provided by private health insurance companies. Private health insurers are obliged to cover a standardised list of services as defined by law, ensuring that every insured person has access to essential healthcare. Insured persons are free to choose and change their health insurance. The deductible is a pre-determined amount that the insured person must pay out-of-pocket for arising medical expenses. If the insured person chooses a higher deductible, this typically results in lower monthly health insurance premiums. Once the deductible has been met, the health insurance company pays for all healthcare and treatment costs, except for an excess of 10 per cent of the costs, which is capped. Voluntary supplementary insurances cover additional benefits.

Generally, pediatricians in private practice are responsible for basic pediatric care. For after-hours care or in critical situations, patients/guardians can contact information hotlines such as the Kids Line or visit directly emergency care facilities, including general urgent care centers or the emergency departments of children's hospitals.

**Supplementary Information 2**

A subgroup analysis was conducted focusing on the safety of patients with TD “Telecare” classified as undertriage from the telemedical perspective, independent of the time interval between teleconsultation and HTS’s evaluation.

Results: This subgroup analysis (n=40) revealed a median time interval between teleconsultation and HTS evaluation of 4.8h (IQR 1.7-11.5h; no hospitalisations, 3 out of 3 indicated improvements in health conditions in the patient feedback).

**Supplementary Information 3**

**List of questions included in the feedback questionnaire sent to the patients 2-3 days after teleconsultation:**

-What would you have done if the option of calling the "Kids Line" would not have existed? (Answer options: I would have taken my child to the emergency department; I would have taken my child to the pediatrician; We would have stayed at home; I don’t know)

-What did you decide to do after the telemedical consultation? (Answer options: I took my child to the emergency department; I took my child to the pediatrician; We stayed at home; plus option for free text to explain the decision)

-How has the health condition of your child developed in the last days? (Answer options: The health condition has improved; The health condition has remained the same; The health condition has deteriorated)

-How satisfied were you with the (administrative) process in the run-up to the telemedical consultation? (6-point Likert scale: very satisfied – very dissatisfied)

-How likely is it that you will recommend the "Kids Line" to others (Likelihood to recommend: 0 (not at all) to 10 (very likely))?

-How is your trust in telemedical consultation? (6-point Likert scale: very high – very low)

**Note:**

-It was not mandatory to answer all the questions (intention: n=601, adherence: n=606, satisfaction: n=606, health condition: n=600).

# Supplementary Tables

**Supplementary Table 1 Patient and call characteristics of the cases of each triage disposition group, along with the results of the statistical tests demonstrating the association of the respective variables with triage disposition.** Data are represented as count and percentage or median (interquartile range), where appropriate. Percentages in parentheses indicate the proportion of each outcome within the respective subgroup (row percentages). Significant differences are marked: *p<0.05.

|  | **Urgent**  **(n=1348)** | **Non-urgent (n=506)** | **Telecare**  **(n=2207)** | **p-value** |
| --- | --- | --- | --- | --- |
| **Age,** *years;* *median (IQR)* | 2.0 (0.0-5.0) | 3.0 (1.0-6.0) | 2.0 (0.0-4.0) | <0.001* |
| **Age categories** |  |  |  | <0.001* |
| *[0-4 months]; n (%)* | 198 (55.9%) | 27 (7.6%) | 129 (36.4%) |  |
| *[5 months – 1 year); n (%)* | 232 (29.6%) | 68 (8.7%) | 484 (61.7%) |  |
| *[1 year – 6 years); n (%)* | 626 (30.0%) | 270 (12.9%) | 1193 (57.1%) |  |
| *[6 years – 12 years); n (%)* | 222 (34.0%) | 109 (16.7%) | 322 (49.3%) |  |
| *[12 years – 18 years); n (%)* | 70 (38.7%) | 32 (17.7%) | 79 (43.6%) |  |
| **Sex** |  |  |  | 0.10 |
| *Female* | 651 (34.4%) | 247 (13.1%) | 994 (52.5%) |  |
| *Male* | 697 (32.1%) | 259 (11.9%) | 1213 (55.9%) |  |
| **Reason for encounter group** |  |  |  | <0.001* |
| *Gastrointestinal complaints; n (%)* | 276 (31.8%) | 81 (9.3%) | 511 (58.9%) |  |
| *Fever; n (%)* | 235 (32.3%) | 97 (13.3%) | 396 (54.4%) |  |
| *Trauma; n (%)* | 272 (39.1%) | 85 (12.2%) | 339 (48.7%) |  |
| *Respiratory complaints; n (%)* | 215 (32.2%) | 97 (14.5%) | 355 (53.2%) |  |
| *Worries; n (%)* | 57 (30.2%) | 23 (12.2%) | 109 (57.7%) |  |
| *Ear pain; n (%)* | 68 (41.2%) | 30 (18.2%) | 67 (40.6%) |  |
| *Dermatological complaints; n (%)* | 36 (15.1%) | 43 (18.0%) | 160 (66.9%) |  |
| *Other*^#^*; n (%)* | 189 (37.1%) | 50 (9.8%) | 270 (53.0%) |  |
| **Day of call** |  |  |  | <0.001* |
| *Working day; n (%)* | 708 (34.3%) | 287 (13.9%) | 1067 (51.7%) |  |
| *Non-working day; n (%)* | 640 (32.0%) | 219 (11.0%) | 1140 (57.0%) |  |
| **Time category of call** |  |  |  | <0.001* |
| *Day shift: 07:00-22:59; n (%)* | 1067 (31.8%) | 476 (14.2%) | 1815 (54.1%) |  |
| *Night shift: 23:00-06:59; n (%)* | 281 (40.0%) | 30 (4.3%) | 392 (55.8%) |  |
| **Qualification of agent** |  |  |  | <0.001* |
| *Pediatric nurses; n (%)* | 633 (28.5%) | 378 (17.0%) | 1207 (54.4%) |  |
| *Pediatricians; n (%)* | 107 (33.1%) | 27 (8.4%) | 189 (58.5%) |  |
| *Non-pediatricians; n (%)* | 608 (40.0%) | 101 (6.6%) | 811 (53.4%) |  |
| ^#^The Reason for encounter group “Other” includes patients with allergies, chest pain, intoxication, neurological problems, urinary tract infection, restlessness, conjunctivitis, or general health issues. | | | | |

**Supplementary Table 2 Baseline characteristics of patients who consulted the Kids Line of the six participating PEDs and whose PED presentation was evaluated by a hospital triage specialist (n=556). b) Characteristics of the corresponding calls.** Data are represented as count and percentage or median (interquartile range), where appropriate. Percentages in parentheses indicate the proportion of each subgroup within the overall cohort (column percentages).

| **a) Baseline characteristics** | **Total**  **(n=556)** |
| --- | --- |
| **Age,** *years;* *median (IQR)* | 2.0 (0.0-5.0) |
| **Age categories** |  |
| *[0-4 months]; n (%)* | 65 (11.7%) |
| *[5 months – 1 year); n (%)* | 96 (17.3%) |
| *[1 year – 6 years); n (%)* | 270 (48.6%) |
| *[6 years – 12 years); n (%)* | 94 (16.9%) |
| *[12 years – 18 years); n (%)* | 31 (5.6%) |
| **Sex** |  |
| *Female; n (%)* | 265 (47.7%) |
| *Male; n (%)* | 291 (52.3%) |
| **Reason for encounter group** |  |
| *Gastrointestinal complaints; n (%)* | 122 (21.9%) |
| *Fever; n (%)* | 89 (16.0%) |
| *Trauma; n (%)* | 124 (22.3%) |
| *Respiratory complaints; n (%)* | 90 (16.2%) |
| *Worries; n (%)* | 18 (3.2%) |
| *Ear pain; n (%)* | 26 (4.7%) |
| *Dermatological complaints; n (%)* | 16 (2.9%) |
| *Other*^#^*; n (%)* | 71 (12.8%) |
| **b) Call characteristics** | **Total**  **(n=556)** |
| **Day of call** |  |
| *Working day; n (%)* | 273 (49.1%) |
| *Non-working day; n (%)* | 283 (50.9%) |
| **Time of call** |  |
| *Day shift: 07:00-22:59; n (%)* | 452 (81.3%) |
| *Night shift: 23:00-06:59; n (%)* | 104 (18.7%) |
| **Qualification of agent** |  |
| *Pediatric nurses; n (%)* | 241 (43.3%) |
| *Pediatricians; n (%)* | 51 (9.2%) |
| *Non-pediatricians; n (%)* | 264 (47.5%) |
| ^#^ The Reason for encounter group “Other” includes patients with allergies, chest pain, intoxication, neurological problems, urinary tract infection, restlessness, conjunctivitis, or general health issues. | |

**Supplementary Table 3 Patient and call characteristics of the cases where the hospital and telemedical perspective agreed and disagreed, along with the results of the statistical tests demonstrating the association of the respective variables with agreement/disagreement of the reviewers.** Data are represented as count and percentage or median (interquartile range), where appropriate. Percentages in parentheses indicate the proportion of each outcome within the respective subgroup (row percentages). Significant differences are marked: *p<0.05.

|  | **Agreement**  **(n=481)** | **Disagreement (n=75)** | **p-value** |
| --- | --- | --- | --- |
| **Age,** *years;* *median (IQR)* | 2.0 (0.0-5.0) | 2.0 (0.5-4.5) | 0.95 |
| **Age categories** |  |  | 0.21 |
| *[0-4 months]; n (%)* | 61 (93.8%) | 4 (6.2%) |  |
| *[5 months – 1 year); n (%)* | 81 (84.4%) | 15 (15.6%) |  |
| *[1 year – 6 years); n (%)* | 228 (84.4%) | 42 (15.6%) |  |
| *[6 years – 12 years); n (%)* | 82 (87.2%) | 12 (12.8%) |  |
| *[12 years – 18 years); n (%)* | 29 (93.5%) | 2 (6.5%) |  |
| **Sex** |  |  | 0.66 |
| *Female* | 231 (87.2%) | 34 (12.8%) |  |
| *Male* | 250 (85.9%) | 41 (14.1%) |  |
| **Reason for encounter group** |  |  | 0.02* |
| *Gastrointestinal complaints; n (%)* | 96 (78.7%) | 26 (21.3%) |  |
| *Fever; n (%)* | 83 (93.3%) | 6 (6.7%) |  |
| *Trauma; n (%)* | 108 (87.1%) | 16 (12.9%) |  |
| *Respiratory complaints; n (%)* | 78 (86.7%) | 12 (13.3%) |  |
| *Worries; n (%)* | 14 (77.8%) | 4 (22.2%) |  |
| *Ear pain; n (%)* | 26 (100.0%) | 0 (0.0%) |  |
| *Dermatological complaints; n (%)* | 13 (81.3%) | 3 (18.8%) |  |
| *Other*^#^*; n (%)* | 63 (88.7%) | 8 (11.3%) |  |
| **Day of call** |  |  | 0.30 |
| *Working day; n (%)* | 232 (85.0%) | 41 (15.0%) |  |
| *Non-working day; n (%)* | 249 (88.0%) | 34 (12.0%) |  |
| **Time category of call** |  |  | 0.11 |
| *Day shift: 07:00-22:59; n (%)* | 396 (87.6%) | 56 (12.4%) |  |
| *Night shift: 23:00-06:59; n (%)* | 85 (81.7%) | 19 (18.3%) |  |
| **Qualification of agent** |  |  | 0.86 |
| *Pediatric nurses; n (%)* | 210 (87.1%) | 31 (12.9%) |  |
| *Pediatricians; n (%)* | 43 (84.3%) | 8 (15.7%) |  |
| *Non-pediatricians; n (%)* | 228 (86.4%) | 36 (13.6%) |  |
| ^#^The Reason for encounter group “Other” includes patients with allergies, chest pain, intoxication, neurological problems, urinary tract infection, restlessness, conjunctivitis, or general health issues. | | | |

**Supplementary Table 4 Baseline characteristics of patients who consulted the Kids Line of the six participating PEDs and filled out the feedback questionnaire (n=606). b) Characteristics of the corresponding calls**. Data are represented as count and percentage or median (interquartile range), where appropriate. Percentages in parentheses indicate the proportion of each subgroup within the overall cohort (column percentages).

| **a) Baseline characteristics** | **Total**  **(n=606)** |
| --- | --- |
| **Age,** *years;* *median (IQR)* | 3.0 (1.0-6.0) |
| **Age categories** |  |
| *[0-4 months]; n (%)* | 44 (7.3%) |
| *[5 months – 1 year); n (%)* | 103 (17.0%) |
| *[1 year – 6 years); n (%)* | 299 (49.3%) |
| *[6 years – 12 years); n (%)* | 119 (19.6%) |
| *[12 years – 18 years); n (%)* | 41 (6.8%) |
| **Sex** |  |
| *Female; n (%)* | 291 (48.0%) |
| *Male; n (%)* | 315 (52.0%) |
| **Reason for encounter group** |  |
| *Gastrointestinal complaints; n (%)* | 113 (18.6%) |
| *Fever; n (%)* | 102 (16.8%) |
| *Trauma; n (%)* | 112 (18.5%) |
| *Respiratory complaints; n (%)* | 92 (15.2%) |
| *Worries; n (%)* | 33 (5.4%) |
| *Ear pain; n (%)* | 21 (3.5%) |
| *Dermatological complaints; n (%)* | 39 (6.4%) |
| *Other*^#^*; n (%)* | 94 (15.5%) |
| **b) Call characteristics** | **Total**  **(n=606)** |
| **Day of call** |  |
| *Working day; n (%)* | 287 (47.4%) |
| *Non-working day; n (%)* | 319 (52.6%) |
| **Time of call** |  |
| *Day shift: 07:00-22:59; n (%)* | 498 (82.2%) |
| *Night shift: 23:00-06:59; n (%)* | 108 (17.8%) |
| **Qualification of agent** |  |
| *Pediatric nurses; n (%)* | 272 (44.9%) |
| *Pediatricians; n (%)* | 51 (8.4%) |
| *Non-pediatricians; n (%)* | 283 (46.7%) |
| ^#^ The Reason for encounter group “Other” includes patients with allergies, chest pain, intoxication, neurological problems, urinary tract infection, restlessness, conjunctivitis, or general health issues. | |
